# Effect of C-reactive protein deficiency on insulin resistance reversal in rats with polycystic ovary syndrome through augmented leptin action

**DOI:** 10.1186/s13098-023-01155-1

**Published:** 2023-09-02

**Authors:** Ke Li, Lingling Hu, Xinrun Li, Zhibin Yuan, Jia He, Dongfang Liu, Gangyi Yang, Lei Yuan

**Affiliations:** 1 Department of Endocrinology, the Second Affiliated Hospital, Chongqing Medical University, Chongqing, 400010 China; 2 Department of general surgery, People’s Hospital, Hubei University of Medicine, Xiangyang No.1, Xiangyang, 441100 China

**Keywords:** C-reactive protein, Leptin, Polycystic ovary syndrome

## Abstract

**Background:**

C-reactive protein(CRP), is an inflammatory marker that weaken leptin bioavailability and insulin sensitivity to disturb energy and glucose metabolism. Polycystic ovary syndrome (PCOS) exhibit a metabolic component consisting of higher plasma CRP levels, hyperinsulinemic and hyperleptinemia. The ability of leptin to regulation of hepatic glucose production (HGP) in the absence of CRP in PCOS remain unknown.

**Methods:**

Dehydroepiandrosterone (DHEA) was used to induce PCOS in rats. We assessed the effects of CRP gene knockout in PCOS model rats on body weight, energy expenditure glucose metabolism and insulin sensitivity. We conducted experiments involving the administration of leptin to both the peripheral and central systems in PCOS model rats with CRP knockout, and studied the effects on changes in glucose kinetics during hyperinsulinemic-euglycemic clamps.

**Results:**

In female PCOS rats, the lack of CRP resulted in decreased leptin resistance and weight gain, increased energy expenditure, and improved insulin sensitivity. Additionally, the deletion of the CRP gene strengthened the HGP-lowering effects of leptin when administered peripherally or centrally. This effect was accompanied by a decrease in the expression of hepatic gluconeogenic enzymes and an increase in hepatic insulin signaling. Finally, inhibition of glucose production was also enhanced for central leptin administration during lipid infusion in PCOS rats.

**Conclusions:**

Our findings highlight the therapeutic potential of targeting CRP to restore glucose homeostasis and insulin sensitivity for leptin in PCOS.

**Supplementary Information:**

The online version contains supplementary material available at 10.1186/s13098-023-01155-1.

## Introduction

Polycystic ovary syndrome (PCOS) is the most common complicated endocrine disease in women of childbearing age. It is characterized by hyperandrogenism, usually associated with hyperinsulinemia and insulin resistance (IR), and often accompanied by morphological manifestations of polycystic ovaries [1]. Women with PCOS often have irregular menstrual cycles, infertility, obesity, hirsutism or acne. The incidence rate of PCOS in women of childbearing age is approximately 6–10% [2]. The rising prevalence rate is the main cause of infertility in women of childbearing age. In addition, women who have polycystic ovary syndrome are recognized to have an elevated risk of experiencing various metabolic, obstetric, psychological and even tumor-related complications in their lifetime, posing a great threat to women’s health [3].

However, the pathophysiological mechanism underlying PCOS remain unclear. Interestingly, chronic low-grade inflammation is recognized as a significant contributor to the development and occurrence of PCOS. PCOS is also a chronic inflammatory mediator disorder [4]. It is well known that C-reactive protein (CRP) is a commonly used and dependable acute-phase reactant inflammation biomarker in clinical. Reports have shown that patients with polycystic ovary syndrome exhibit elevated levels of CRP, and this indicator is associated with infertility as well as diabetes [5] and cardiovascular disease [6]. These findings imply that CRP may play a multiple action in PCOS patients [7]. Although these strong correlations have been found in clinical studies, the current research is limited to the observation of the PCOS population. Up to now, there are no reports on the pathophysiological mechanism of CRP in the progression of PCOS and whether CRP affects insulin resistance, glucose tolerance, and even hepatic glucose flux in PCOS.

Leptin is a peptide composed of 167 amino acids that is secreted by adipose tissue. As an adipokine, leptin has important effects on food intake, energy storage and body weight. The development of obesity, insulin resistance, and type 2 diabetes is closely associated with the significant feature of leptin resistance [8]. Previous studies have shown that patients with PCOS have hyperleptinemia, which is positively correlated with obesity, insulin and body mass index [9]. Because hyperleptinemia often reflects the existence of leptin resistance, it can be inferred that patients with PCOS also have leptin resistance. Studies have found that in insulin-resistant people, binding of human C-reactive protein (CRP) to leptin inhibits leptin signaling and impairs its function, thus exacerbating leptin resistance [10]. Our previous studies have shown that CRP can directly regulate energy balance, body weight, insulin sensitivity, and glucose homeostasis by affecting leptin’s central effects in the hypothalamus [11].

PCOS is a metabolism-related disease that integrates chronic low-grade inflammation, insulin resistance and leptin resistance [9]. CRP concentrations are elevated in women with PCOS [12] and has been closely linked to the insulin resistance. Collectively, these evidences suggest that CRP may exert some of its metabolic actions in part via interaction with leptin in PCOS. Accordingly, we used CRP knockout (KO) rats to construct a PCOS model to explore whether CRP gene deletion affects insulin resistance, hepatic glucose homeostasis and leptin bioactivity in PCOS rats. We hypothesize that the amplification of leptin action to lower hepatic glucose production (HGP) and promote insulin sensitivity in the absence of CRP in individuals with the hyperandrogen environment.

## Materials and methods

### Animal experiments

Animal experiments were performed according to protocols approved by the Animal Care Committee of Chongqing Medical University. CRP gene knockout rats were donated by Professor Zhao Zijian of Nanjing Medical University and reproduced in the Animal Experiment Center of Chongqing Medical University. Detailed production method as described previously [11]. The animals were housed in a facility equipped with temperature and humidity control, following a 12-hour light/dark cycle. They were provided with ad libitum access to food and water. The animals were randomly allocated to different treatment groups.

### Rat models

A total of 150 4-week-old female rats, either CRP knockout or littermate wild-type, were randomly assigned to either a normal chow diet (ND) or a high-fat diet (HFD) feeding group. Additionally, the rats were randomly allocated to different treatment groups. Female rats were injected daily subcutaneous with dehydroepiandrosterone (DHEA) (Sigma Aldrich) at a dose of 60 mg/kg/day [13] dissolved in 0.2 ml sesame oil for up to 90 days, while the control group were injected with 0.2 ml sesame oil for an equivalent length of time. Vaginal exfoliative cytology was performed for 10 consecutive days on the 20th day after the start of subcutaneous administration, and the rats that exhibited successful development of the PCOS pathology were chosen for follow-up experiments.

### Light microscope analysis

The ovarian tissues of rats were fixed in 4% paraformaldehyde for 24 h, thereafter, they were embedded in paraffin wax. The paraffin-embedded ovary Sect. (4 𝜇m) were stained using hematoxylin and eosin and calculated the number of follicles using a light microscope as described previously [14].

### Third ventricle and jugular arteriovenous cannulations

The stereotaxic brain surgeries were performed 7 days before the jugular arteriovenous cannulations. Indwelling catheters were stereotactically inserted into the third ventricle of the brain. Correct intubation position was confirmed as described previously [15]. For the euglycemic hyperinsulinemic clamp (EHC) study, indwelling catheters were inserted into the internal jugular vein and carotid artery for infusion and blood sampling seven days after stereotactic surgery. Recovery from the surgery was monitored for a four-day period by measuring food intake and weight.

### Metabolic analyses

Daily body weight measurements were taken from rats aged 4–16 weeks. Food intake and rectal temperature were recorded at specific time points and durations. The rectal temperature was measured using an electronic thermometer. Energy expenditure was determined by measuring the 24-hour rates of VO2 and VCO2 production, as described previously [11].

### Intraperitoneal glucose and insulin tolerance experiments

Following a 6-hour fast, rats received an intraperitoneal injection of 25% glucose (2.0 g/kg) for the glucose tolerance test (GTT) or insulin (1.0 U/kg, Novolin R, Nordisk, Bagsvaerd, Denmark) for the insulin tolerance test (ITT). Venous blood was collected at the designated time before and after injection in order to quantitatively assess levels of glucose and insulin.

### EHC and intracerebroventricular (ICV) infusion

Following a 12-hour fast, EHC was performed while the subjects were conscious. In brief, a continuous infusion of [3-H^3^] glucose that had undergone high-performance liquid chromatography (HPLC) purification (PerkinElmer, Waltham, MA) was started at t = 0 min with a 6 µCi bolus followed by a 0.2 µCi/min rate of infusion, which continued for a total of 240 min. The EHC was started at 120 min after the tracer infusion. Somatostatin (3 µg/kg/min) was infused intravenously together with insulin (6.0 mU/kg/min) to inhibit endogenous insulin secretion. A variable infusion of 25% glucose was started and adjusted every 10 min to maintain blood glucose at the baseline level. Subgroups of rats undergoing intravenous or ICV administration of leptin procedures, recombinant rat leptin (0.5ug/kg/min for peripheral, 0.83 µg/h for central, R&D Systems) was initiated at t = − 120 and continued until the end of the clamp at t = 240 min. To determine insulin, free fatty acids (FFA), and glucose-specific activity, blood samples were collected from the jugular vein catheter at 0, 120, 200, 220, 230, and 240 min. Subsequently, the rats were anesthetized, and tissue samples were freeze-clamped in situ using aluminum tongs pre-cooled with liquid nitrogen and stored at -80ºC for further analysis.

### Biochemical analysis

Plasma glucose was measured using the glucose oxidase method. Plasma insulin was measured using a commercial RIA kit (HTA CO.LTD. Beijing, China). Plasma levels of leptin, CRP, estradiol (E2) ,testosterone (T), progesterone (P), follicle stimulating hormone (FSH) and luteinizing hormone (LH) were detected by commercial enzyme-linked immunosorbent assay kits (Jingmei Biotechnology, Yancheng, China). [3-^3^ H] Glucose radioactivities was detected by scintillation counter.

### mRNA analysis

To measure the expression levels of mRNA, we used Quantitative Real-Time-PCR (qRT-PCR). We analyzed mRNA expressions using the comparative threshold cycle method and normalized with β-actin. The following primers were used: 5’-CCCTGAACCCTAAGGCCAACCGTGAAAA-3’ and 5’-TCTCCGG AGTCCATCACAA TGCCTGTG-3’ for β-actin, and 5’-AGTCACCATCACTTCCTGGAAGA-3’ and 5’-GGTGCAGAATCGCGAGTT-3’ for phosphoenolpyruvate carboxykinase (PEPCK).

### Western blot analyses

The tissue samples were homogenized and the protein levels were determined using a BCA quantification kit (Beyotime Biotechnology, Shanghai, China). The protein lysates underwent 8% SDS-PAGE and were transferred to polyvinylidene difluoride membranes. Following this, the membranes were probed with 1:1,000 diluted primary antibodies against the insulin receptor (InsR) / phospho-InsR, AKT kinase (AKT) / phospho- AKT (Cell Signaling Technology, Beverly, MA, USA), PEPCK (Santa Cruz Biotechnology, Dallas, TX, USA); and GAPDH (Abcam, Cambridgeshire, UK) at 4 °C. The membranes were washed three times with Tris-buffered saline and then incubated with a horseradish peroxidase-labeled sheep anti-rabbit antibody for 1 h. The blots were visualized using enhanced chemiluminescence.

### Statistical analysis

All data are expressed as means ± SEM. Sample size was determined using PASS (version 15.0). Statistical analysis was performed using SPSS (version 21.0). The normality of data distribution was assessed using the Shapiro-Wilk test. Then, our results were analyzed by an unpaired Student’s t-test when comparing two groups, or by ANOVA followed by Tukey’s post hoc test for more than two groups. *P* < 0.05 was indicated significant.

## Results

### DHEA induced the PCOS model in rats

After the DHEA treatment, rats lost their regular estrous cycles and stayed in the anoestrus period, smears showed the presence of a large number of white blood cells (Supplementary Fig. 1D), while few keratinized cells and epithelial cells were visible, suggesting anovulation. In contrast, the control group had normal estrous cycles (Supplementary Fig. 1A-D). Furthermore, pathological morphology showed the number of cystic follicles increased with cystic expansion and the number of granulosa cell layers was reduced in DHEA-treated group. However, ovarian tissue in control group showed orderly arranged granulosa cells and morphologically integrity (Supplementary Fig. 1E). We also measured the sex hormone concentration in serum. Compared with the control group, the levels of estradiol (E2), testosterone (T), luteinizing hormone (LH) and the rate of luteinizing hormone/follicle stimulating hormone (LH/FSH) in PCOS group were significantly increased, while the level of progesterone (P) was significantly reduced (Supplementary Table 1). According to Rotterdam PCOS diagnostic criteria [1], we established a PCOS rat model successfully.

### Increase in CRP and leptin in PCOS model rats

The expression level of leptin in PCOS group were significantly higher than that in control group. There were almost no CRP expression in CRP KO groups. Interestingly, CRP were up-regulated in the DHEA wild-type (WT) group compared with that of the non-DHEA-WT group. Compared with ND-CRP KO group, Leptin in the HFD-DHEA-CRP KO group was significantly increased (Table 1).

**Table 1:** Serum leptin and CRP of the rat in different groups

	Leptin (ng/ml)	CRP (ng/ml)
ND-WT	4.84 ± 1.55	278.26 ± 38.77
ND-CRP KO	3.56 ± 0.81	4.68 ± 1.04
ND-DHEA-WT	9.27 ± 2.30^*^	368.92 ± 72.05^*^
ND-DHEA-CRP KO	5.83 ± 1.16	5.72 ± 2.30
HFD-WT	6.86 ± 1.05	326.51 ± 32.95
HFD-CRP KO	4.21 ± 1.12	4.52 ± 1.24
HFD-DHEA-WT	10.38 ± 2.20^**^	397.51 ± 56.00^*^
HFD-DHEA-CRP KO	6.30 ± 2.06^#^	6.21 ± 1.65

ND, normal chow diet; HFD, high fat diet;DHEA ,dehydroepiandrosterone. ^*^P < 0.05, ^**^P < 0.01 vs. ND-WT group, ^#^*P* < 0.05 vs. ND-CRP KO group

### Effect of CRP lack on energy metabolism in PCOS rats

**Fig. 1:**
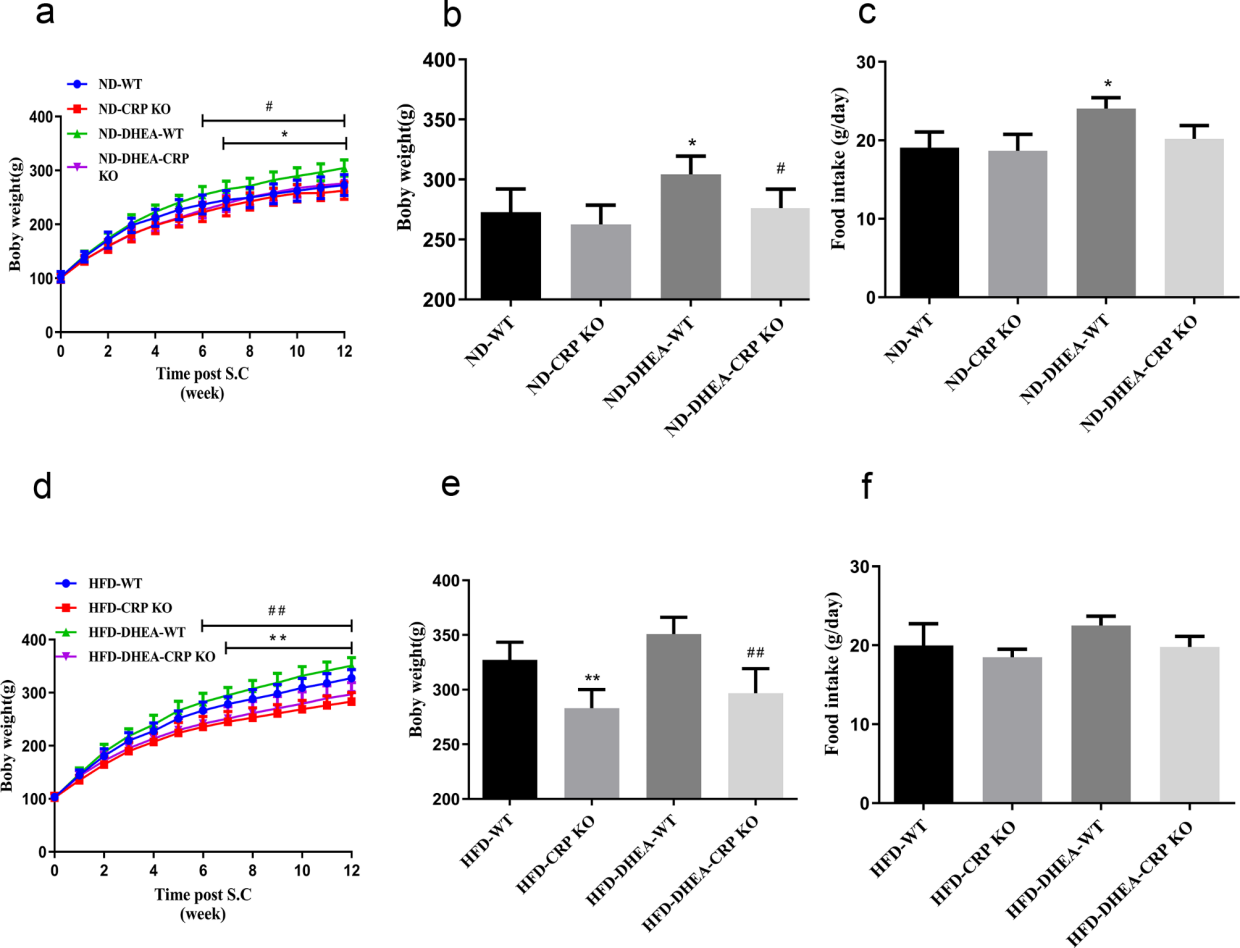
Changes in body weight and food intake in 4-month-old female rats. Four-week-old of placebo or dehydroepiandrosterone (DHEA)-treated female WT and CRP KO rats were fed a ND or HFD for 3 month. **(a, d)** Body weight gain curve. **(b, e)** Cumulative body weight. **(c ,f)** Average daily food intake. Data are expressed as the mean ± SEM (n = 6 rats for each group). ND, normal chow diet; HFD, high-fat diet. ^*^P < 0.05 vs. ND-WT group; ^#^*P* < 0.05 vs. ND-DHEA-WT group; ^**^P < 0.05 vs. HFD-WT group, ^##^*P* < 0.05 vs. HFD-DHEA-WT group

To examine the impact of CRP deficiency on energy homeostasis in individuals with PCOS from a physiological perspective, we monitored the heart rate, blood pressure, body weight, food intake. First of all, there was no statistically significant difference in heart rate among the rats in each group. Compared with the non-DHEA group, the systolic and diastolic blood pressure of the DHEA group were significantly higher (Supplementary Table 2). Interestingly, CRP KO rats exhibit a significant resistance to the increase in systolic and diastolic blood pressure induced by HFD or DHEA treatment (Supplementary Table 2). When rats fed a normal chow diet, the body weight of the DHEA group was significantly higher than that of the non-DHEA group, but there was no significant difference between the DHEA group and the non-DHEA group in CRP KO rats (Fig. 1a, b). Interestingly, the body weight of the ND-DHEA-CRP KO group was lower than that of the ND-DHEA-WT group. After an HFD regimen, the body weight of CRP KO rats were significantly reduced no matter with or without DHEA-treated compared with WT rats (Fig. 1d, e). However, there was no significant difference in body weight between the PCOS model group and the non-PCOS model group. The mean daily food intake was higher in ND-DHEA-WT group than in the ND-WT group (Fig. 1c), while there was no significant difference in food intake between groups of HFD-fed rats (Fig. 1f).

**Fig. 2:**
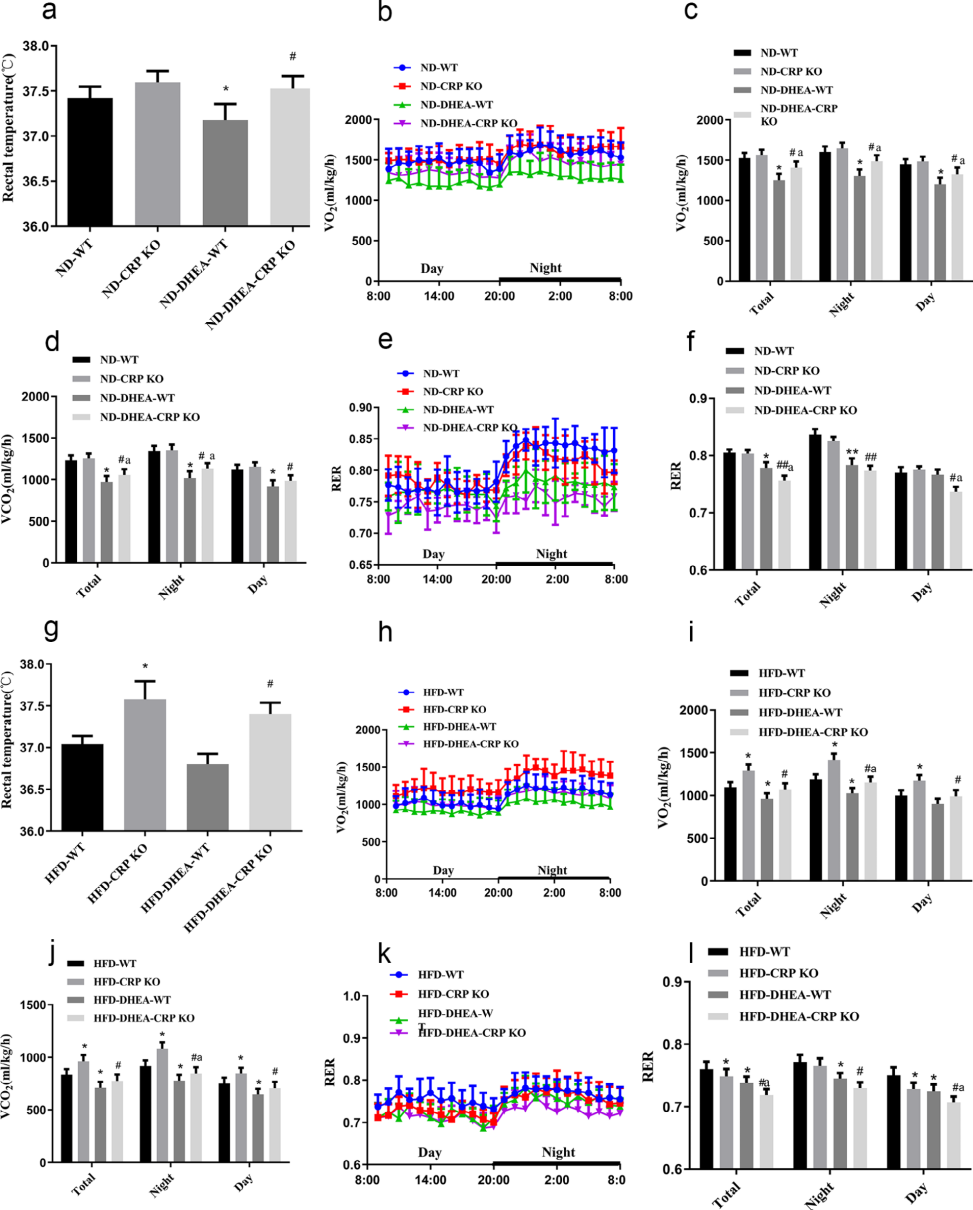
PCOS rats showed lower energy expenditure, while CRP KO rats showed increased energy expenditure in PCOS and HFD. CRP KO rats showed an increase in rectal temperature under DHEA-treated **(a)** or high-fat feeding **(g)** (^*^P < 0.05 vs. WT group; ^#^*P* < 0.05 vs. DHEA-WT group). In metabolic cage studies, both 24 h V_O2_**(b, c, h, i)** and 24 h V_CO2_**(d, j)** were all significantly increased, while the RER ratios **(e, f, k, l)** were significantly decreased in CPR KO rats treated with DHEA or fed with HFD. (^*^P < 0.05 vs. WT group; ^#^*P* < 0.05, ^##^*P* < 0.01 vs. CRP KO group, ^a^*P*<0.05 vs. DHEA-WT group). Data are expressed as the mean ± SEM (n = 5 rats for each group)

To further analyze the effect of CRP lack on energy expenditure in PCOS rats, we also examined the anal temperature and respiratory quotient. Our results showed that the energy consumption of CRP KO rats increased.In line with this observation, following a DHEA-treated or HFD, rectal temperature was higher in CRP KO rats than that in the WT controls (Fig. 2a, g). In a 24-hour metabolic study, when rats maintained on a ND diet, compared with the non-PCOS model groups, the oxygen consumption(V_O2_) (Fig. 2b,c), carbon dioxide production(V_CO2_) (Fig. 2d) and the respiratory exchange rate (RER) (Fig. 2e,f) of the PCOS model rats decreased significantly. However, CPR KO abolished the ability of DHEA-treated to lower V_O2_ and V_CO2_ in partially (Fig. 2b-f). Similar findings were discovered in HFD-fed rats (Fig. 2h-l). When under an HFD-fed regime, V_O2_ (Fig. 2h, i) and V_CO2_ (Fig. 2j) in CRP KO rats were higher compared to those in WT rats, while RER (Fig. 2k, l) was significantly reduced. Taken together, PCOS and HFD-fed rats are more likely to reduce energy expenditure and thus contribute to obesity, but CRP deficiency reverses this process.

### CRP deficiency alleviates insulin resistance in PCOS rats

**Fig. 3:**
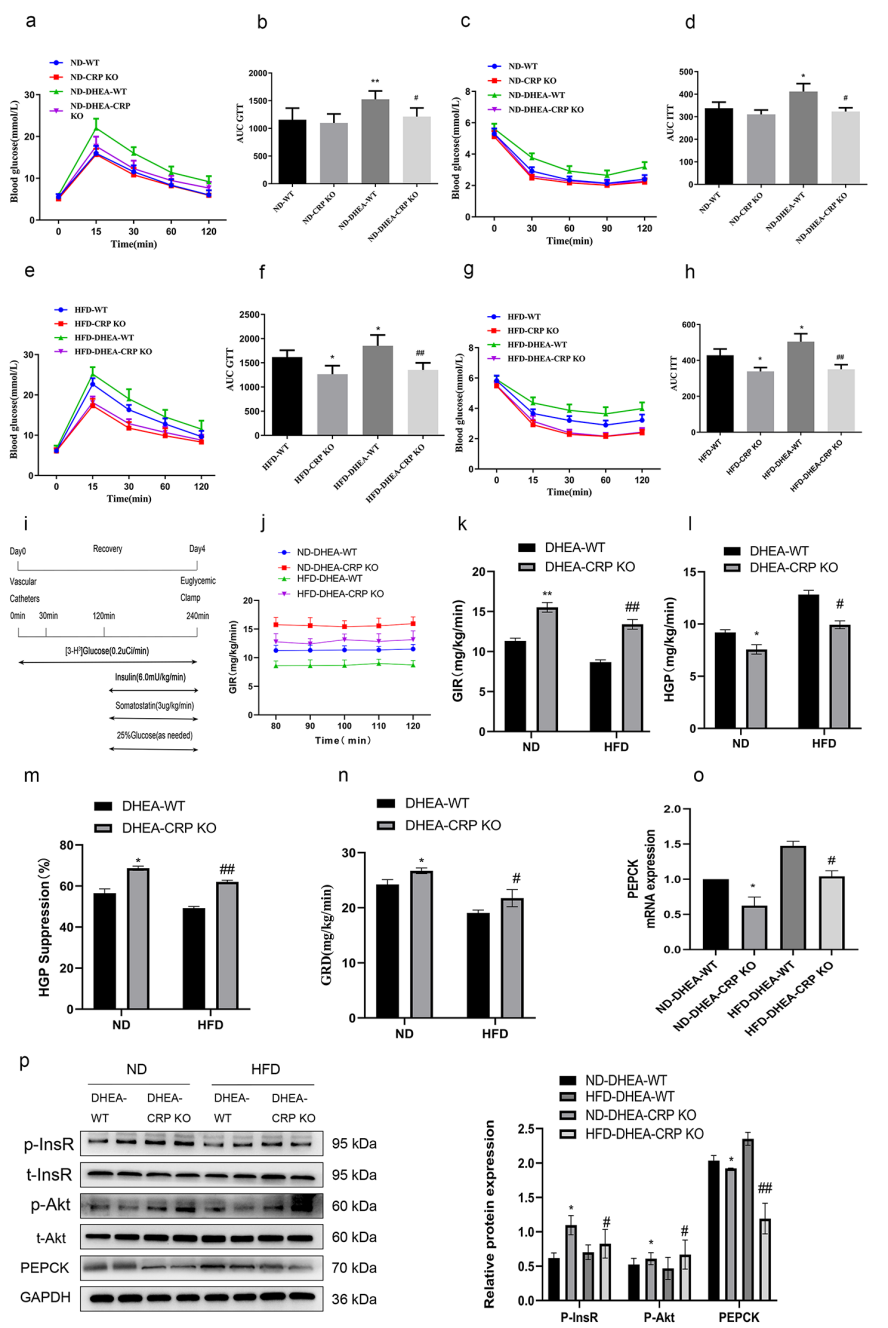
Deficiency of CRP decreases susceptibility to DHEA and HFD-induced insulin resistance. Four-week-old of placebo or dehydroepiandrosterone (DHEA)-treated female WT and CRP KO rats were fed a ND or HFD for 3 month. **(a, e)** Blood glucose levels during the glucose tolerance test (GTT). **(b, f)** Area under curve (AUC) during the GTT. **(c, g)** Blood glucose levels during the insulin tolerance test (ITT). **(d, h)** AUC during the ITT.(^*^P < 0.05, ^**^P < 0.01vs WT group; ^#^*P* < 0.05, ^##^*P* < 0.01 DHEA-WT group) **(i)** Experimental procedure and EHC protocol. **(j)** Glucose infusion rate (GIR) time course. **(k)** Cumulative GIR (l). Hepatic glucose production (HGP). **(m)** Percentage of suppression of HGP. **(n)** Glucose disappearance rates (GRD). **(o)** RT-PCR assays of hepatic PEPCK mRNA **(p)** Immunoblotting assays of hepatic expression of PEPCK protein, phosphorylation of InsR and Akt and densitometric quantification (^*^P < 0.05, ^**^P < 0.01vs ND-DHEA-WT group; ^#^*P* < 0.05, ^##^*P* < 0.01 HFD-DHEA-WT group). Data are expressed as the mean ± SEM (n = 6 rats for each group)

Hyperinsulinemia and insulin resistance are common clinical manifestations of PCOS. Elevated CRP levels are clinically linked to the development of insulin resistance, which can ultimately lead to type 2 diabetes. Therefore, we studied the connection between CRP deficiency and insulin resistance in PCOS rats. Firstly, consistent with previous studies [11], the CRP KO rats exhibited improved GTT and ITT curves compared to the WT rats under HFD feeding (Fig. 3e, g). For another, under the same conditions, the area under curves (AUC) of GTT and ITT in DEHA-treated rats was significantly higher than that of non-DHEA-treated rats, while the AUC of GTT and ITT in CRP KO rats was lower than that of WT controls in PCOS model (Fig. 3b, d, f, h). To evaluate the impact of CRP deficiency in the control of glucose fluxes in PCOS model rats, EHC protocol was performed on conscious rats fed ND or HFD (Fig. 3i). Under the ND or HFD schemes, the glucose infusion rate (GIR) and glucose disposal rate (GRD) of PCOS rats were lower than those of WT rats, while HGP was higher than that of non-PCOS rats (data not shown). Knockout of CRP reversed the ability of DHEA to decrease GIR (Fig. 3j, k), GRD (Fig. 3n) and elevate glucose production (Fig. 3l, m). These results suggest that PCOS rats are associated with a marked decrease in hepatic insulin sensitivity, while the general insulin sensitivity in CRP KO rats is enhanced. Next, we examined expressions of PEPCK and phosphorylated insulin signaling in PCOS rat fed with ND or HFD. Consistent with the results of the clamp study, compared with the WT controls, whether ND or HFD feeding, the expression of PEPCK in the liver of CRP KO rats decreased significantly (Fig. 3o, p), but the phosphorylation level of InsR and Akt in the liver were increased significant (Fig. 3p). To sum up, these observations indicate that CRP directly regulates hepatic insulin sensitivity in PCOS.

### Effect of CRP deficiency on leptin function (peripheral and central) in rats with PCOS

**Fig. 4:**
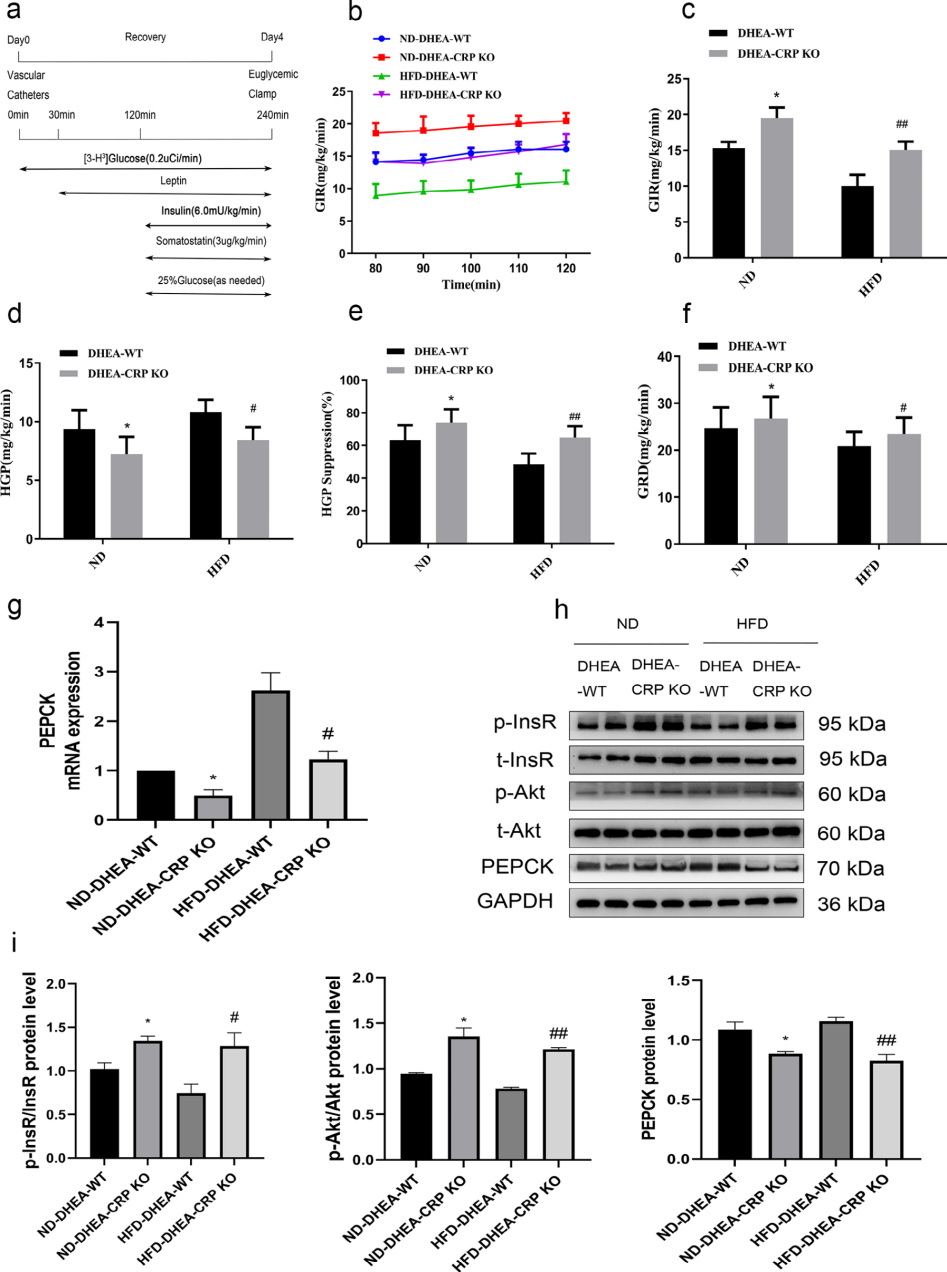
CRP deficiency enhance the role of peripheral leptin on restoring insulin sensitivity in PCOS rats fed a ND or HFD. **(a)** Experimental procedure and EHC protocol. **(b)** GIR time course **(c)** Cumulative GIR. **(d)** Hepatic glucose production (HGP). **(e)** Percentage of suppression of HGP. **(f)** Glucose disappearance rates (GRD). **(g)** RT-PCR assays of hepatic PEPCK mRNA **(h, i)** Immunoblotting assays of hepatic expression of PEPCK protein, phosphorylation of InsR and Akt and densitometric quantification. Data are expressed as the mean ± SEM (n = 5–6 rats for each group). ^*^P < 0.05, ^**^P < 0.01 vs. ND-DHEA-WT group; ^#^*P* < 0.05, ^##^*P* < 0.01 HFD-DHEA-WT group

**Fig. 5:**
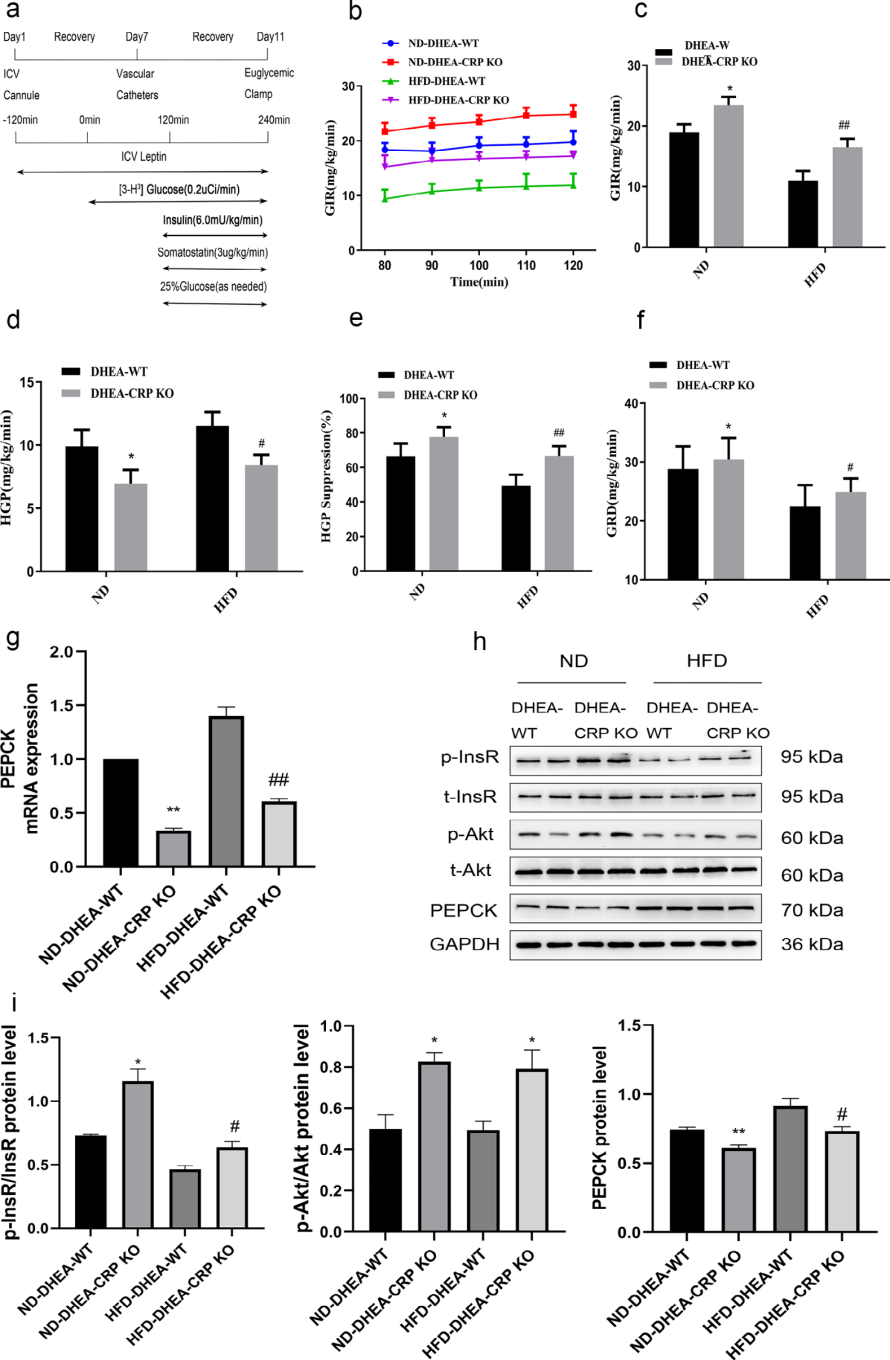
CRP deficiency enhance the role of central leptin on restoring insulin sensitivity in PCOS rats fed a ND or HFD. **(a)** Experimental procedure and EHC protocol. **(b)** GIR time course **(c)** Cumulative GIR. **(d)** Hepatic glucose production (HGP). **(e)** Percentage of suppression of HGP. **(f)** Glucose disappearance rates (GRD). **(g)** RT-PCR assays of hepatic PEPCK mRNA **(h, i)** Immunoblotting assays of hepatic expression of PEPCK protein, phosphorylation of InsR and Akt and densitometric quantification. Data are expressed as the mean ± SEM (n = 5–6 rats for each group). ^*^P < 0.05, ^**^P < 0.01 vs. ND-DHEA-WT group; ^#^*P* < 0.05, ^##^*P* < 0.01 HFD-DHEA-WT group

It is indisputable that leptin plays a crucial role in regulating insulin action and hepatic glucose fluxes. Previous study has demonstrated that the administration of leptin centrally can improve hepatic insulin resistance that is induced by a HFD [16]. On the other hand, report indicated that CRP could binds to leptin and attenuates its physiological functions [10]. To explore the effect of leptin infusion on hepatic glucose output in CRP KO of PCOS rats, we performed peripheral and central injection of leptin in PCOS model rats using an EHC study (Figs. 4a and 5a). When leptin was administered peripherally, whether the rats were maintained on an ND or HFD diet, the GIR (Fig. 4b, c), GRD (Fig. 4f) and hepatic glucose inhibition rates (Fig. 4e) of CRP KO rats were significantly higher than those of WT rats, while HGP (Fig. 4d) was lower than that of WT rats, indicated that leptin inhibited HGP more pronounced in the absence of CRP in PCOS. Similar results were found when leptin was administered in central (Fig. 5a-f, the blood metabolic parameters measured during the EHC can be found in Supplementary Table 3). In line with the results of the clamp study, whether infusion of leptin by peripheral or central, mRNA and protein expression of PEPCK in liver of CRP KO rats were significantly decreased compared with their WT controls, while the phosphorylated InsR and Akt in the livers were significant increased in CRP KO rats (Figs. 4g-i and 5g-i). Collectively these data powerfully indicate that CRP deficiency can improve insulin resistance and enhanced the influence of leptin on hepatic glucose kinetics and insulin signaling in PCOS rats.

### CRP deficiency improve the lipid-induced hepatic insulin resistance during central leptin infusion in PCOS rats

**Fig. 6:**
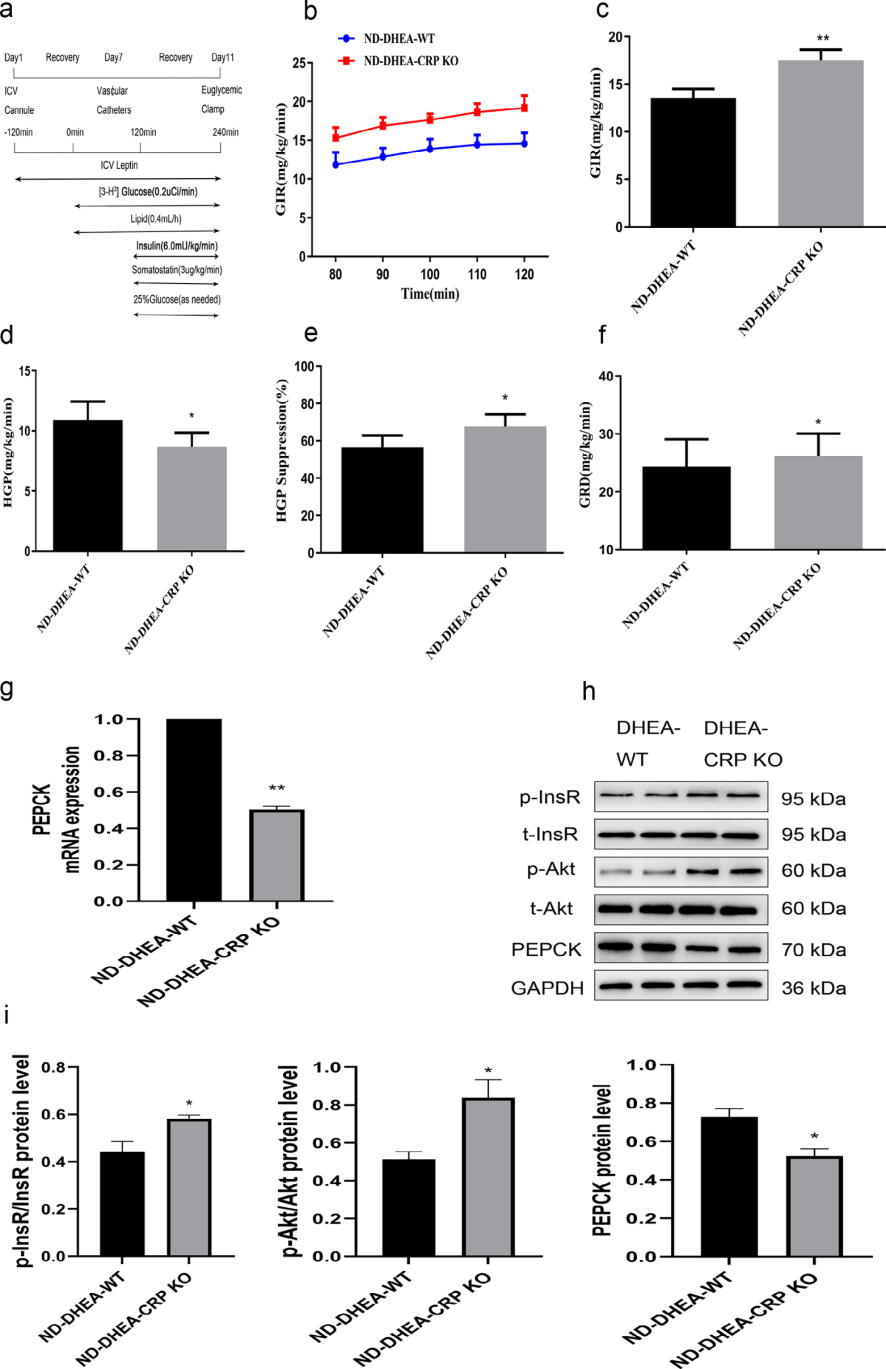
CRP deficiency enhance the role of central leptin on restoring insulin sensitivity during lipid infusion in PCOS rats fed a ND. **(a)** Experimental procedure and EHC protocol. **(b)** GIR time course **(c)** Cumulative GIR. **(d)** Hepatic glucose production (HGP). **(e)** Percentage of suppression of HGP. **(f)** Glucose disappearance rates (GRD). **(g)** RT-PCR assays of hepatic PEPCK mRNA **(h, i)** Immunoblotting assays of hepatic expression of PEPCK protein, phosphorylation of InsR and Akt and densitometric quantification. Data are expressed as the mean ± SEM (n = 5–6 rats for each group). ^*^P < 0.05, ^**^P < 0.01 vs. ND-DHEA-WT group

To further evaluate the effect of central leptin on hepatic glucose flux in the presence of altered nutrition sensing in the periphery for PCOS rats, we constructed an acute insulin resistance model induced by peripheral lipid infusion in ND-fed PCOS rats during the clamps (Fig. 6a). Intriguingly, compared with the WT group, the GIR (Fig. 6b, c), GRD (Fig. 6f) and hepatic glucose inhibition rates (Fig. 6e) of the CRP KO group were significantly higher, and the HGP was significantly lower (Fig. 6d). In addition, immunoblot analysis showed that the phosphorylation levels of InsR and Akt in the liver of mutant rats were more pronounced and the expression levels of PEPCK were significantly reduced than those in the WT rats (Fig. 6g-i). Therefore, CRP deficiency raises the efficacy that central leptin controls hepatic glucose flux and improve hepatic insulin resistance induced by lipid infusion.

## Discussion

Currently, PCOS is believed to be linked with both insulin resistance and hyperinsulinemia, in addition to symptoms such as irregular menstrual cycles and excess levels of androgen. Insulin resistance and unhealthy obesity are thought to contribute significantly to the onset and progression of PCOS. Pathogenic factors such as hypertension, dyslipidemia, hyperglycemia, obesity, and metabolic dysfunction significantly increase the risk of long-term adverse cardiovascular events and type 2 diabetes [17] in PCOS patients. The PCOS model constructed by subcutaneous injection of DHEA showed a disordered oestrus cycle, polycystic formation of ovarian atresia follicles, regression of granulosa cells, disturbed sex hormone levels, and metabolic dysfunction, including weight gain, increased leptin levels and insulin resistance. Moreover, Ressler et al. [18] found that high-fat-fed PCOS model rats showed more severe obesity and insulin resistance and stronger depression. All these phenotypes are extraordinarily similar to the characteristics of PCOS in humans and provide an excellent animal model for studying the ovarian and metabolic disorders of PCOS.

The presence of low-grade chronic inflammation is indicated by having consistently slightly elevated levels of CRP within the normal range, more recently has been linked to PCOS in women. Studies have shown that CRP concentrations are elevated in women with PCOS [12]. Significantly higher serum TNF-α, IL-6, IL-18 [19], plasminogen activator inhibitor-1, as well as higher white blood cell (WBC) counts revealed peripheral inflammation conditions in PCOS [20]. In addition, increased WBC and CRP levels in PCOS have also been shown to be independent of obestiy [21]. The positive correlation between CRP concentrations and HOMA-IR suggests that insulin resistance is linked to low-grade chronic inflammation. Our study found that CRP was significantly increased in both the normal diet and high-fat diet of wild-type PCOS rats, which is similar to what has been found in humans. Increased CRP concentrations may be related to vascular endothelial injury, which can activate the inflammatory response system and stimulate CRP synthesis in the liver. At the same time, CRP can activate inflammatory cells to take up low density lipoprotein so that the formation of foam cells can activate endothelial cells to increase the expression of adhesion factors and inflammatory signals, reduce the production of vascular relaxation factors such as nitric oxide (NO) [22], and promote the proliferation of vascular smooth muscle cells [23]. In our study, the changes in blood pressure of PCOS model rats with CRP gene knockout were not significant, while reverse in wild-type rats. This was consistent with the finding that CRP levels were correlated with hypertension [24].

Our finding that CRP knockout rats had higher anal temperature and oxygen consumption suggests that CRP knockout rats may have a higher basal metabolic rate. CRP knockout PCOS rats had a lower respiratory quotient, suggesting that their metabolic substrates may be more prone to fat, and their adipose tissues may be more sensitive to catecholamines. Catabolism of fat requires the stimulation of hormones such as catecholamines. Fat desensitization to catecholamines leads to a reduction in fat catabolism, resulting in obesity. Leptin can enhance the effect of catecholamines [25]. The association of PCOS patients with adipose tissue catecholamine resistance, insulin resistance and obesity has been well documented [26]. We speculated that the PCOS model constructed by CRP knockout rats may have a higher basal metabolic rate and a lower body weight because the loss of the CRP gene weakened catecholamine resistance in adipose tissue. In addition, studies in skeletal muscle cells have found that CRP can even directly inhibit the activation of insulin signaling. CRP disturb the phosphorylation of insulin receptor substrate 1 (IRS-1) in muscle cells and attenuates insulin signaling, thereby disrupting glucose transport [27]. Several studies in people with impaired glucose tolerance and impaired fasting glucose have verified that CRP is positively correlated with insulin resistance, obesity, and blood triglyceride levels [28]. By constructing the PCOS model, we found that CRP knockout rats showed better glucose disposal ability and higher insulin sensitivity than wild-type rats in GTT and ITT tests, which further reflected that CRP may be implicated in insulin resistance in PCOS.

White adipose tissue is the primary source of leptin production in humans. It’s widely accepted that leptin participates in the regulation of food intake, body mass, lipolysis, energy metabolism, proinflammatory immune responses and even reproductive functioning. After being released from adipocytes into the bloodstream, leptin crosses the blood-brain barrier (BBB) and reaches specific areas of the brain that regulate food intake, energy expenditure, and glucose metabolism [29]. Leptin’s aberrant expression and malfunction are significant factors in the development of PCOS, with insulin being considered as the key regulator of leptin production. Prolonged hyperinsulinemia results in an increase in circulating levels of leptin. Current studies suggest that PCOS patients have hyperleptinemia, the level of which is positively correlated with serum leptin, insulin and body mass index [9]. Indeed, we found that an increase level of leptin in PCOS model rats. In combination with the enlargement of central leptin infusion to lower glucose production and regulate insulin sensitivity in the absence of CRP, we deduce that hypothalamic inflammation may mediate the leptin resistance in PCOS.

A robust positive correlation was observed between plasma hypersensitive CRP and leptin levels in healthy people [30], as well as obese [31] and diabetic patients [32]. It is believed that chronically high levels of CRP aggravate leptin resistance in insulin-resistant people. Tracing it to its cause, circulating CRP and leptin can regulate each other’s bioavailability. On the one hand, leptin can stimulate CRP synthesis in liver cells in a PI3K-dependent manner. Leptin binds to leptin receptors on endothelial cells, activating downstream leptin signaling and increasing CRP expression in the vascular endothelium. On the other hand, CRP forms five polymer molecules in vivo, which bind to leptin and directly affect the affinity between leptin and its receptors, thereby blocking the transmission of downstream leptin signals (including AMPK, AKT, and phosphorylation of endothelial nitric oxide synthase) [33]. CRP in chronic inflammatory conditions of obesity can also damage the function of BBB and increase BBB permeability, resulting in reactive glial proliferation and affecting central nervous system function, which may be a major factor of central leptin resistance in chronic inflammation [34]. Whether leptin was administered by peripheral or central, we found that CRP gene knockout rats have stronger insulin sensitivity to regulate hepatic glucose production. We speculated that peripheral infusion of leptin may successfully cross the blood‒brain barrier and bind to its receptors in the hypothalamus due to CRP deficiency, then improving liver glucose metabolism through the brain-liver axis. These data highlight the vital role of leptin to maintain glucose homeostasis rely on the change of CRP in PCOS.

In summary, our findings provide novel insights into the connection between CRP and leptin in the regulation of hepatic glucose homeostasis and insulin sensitivity in PCOS. This finding may reveal novel therapeutic approach targets to reduce inflammation signaling pathways to restore glucose homeostasis in PCOS.

### Electronic supplementary material

Below is the link to the electronic supplementary material.

Supplementary Material 1

Supplementary Material 2

## Data Availability

The data used in this research can be made available by the corresponding author upon a reasonable request.
