# Supplementary material for: Effect of C-reactive protein deficiency on insulin resistance reversal in rats with polycystic ovary syndrome through augmented leptin action
Source: Diabetol Metab Syndr. 2023 Sep 2;15:180. doi: 10.1186/s13098-023-01155-1 (PMC10474659; doi:10.1186/s13098-023-01155-1)
Supplement: Supplementary file 1 — Supplementary Material 1 [file 13098_2023_1155_MOESM1_ESM.docx]

**Supplementary Table 1. Sex hormones in [different](javascript:;) groups**

|  | E2 (pg/ml) | P (ng/ml) | T (nmol/ml) | LH (mIU/ml) | FSH (mIU/ml) | LH/FSH |
| --- | --- | --- | --- | --- | --- | --- |
| ND-WT | 60.01±9.35 | 19.94±2.56 | 0.95±0.21 | 14.56±1.87 | 8.02±1.62 | 1.82±0.24 |
| ND-CRP KO | 58.18±11.96 | 18.00±2.38 | 0.91±0.23 | 14.42±0.95 | 8.51±1.49 | 1.69±0.56 |
| ND-DHEA-WT | 78.11±11.06^*^ | 14.83±1.69^*^ | 1.90±0.44^*^ | 19.09±2.58^*^ | 5.80±0.67 | 3.29±0.74^*^ |
| ND-DHEA-CRP KO | 73.95±11.51^#^ | 14.11±3.02^#^ | 1.67±0.43^#^ | 18.35±2.29^#^ | 5.65±1.17 | 3.24±1.09^#^ |
| HFD-WT | 61.23±10.09 | 18.67±2.33 | 1.02±0.25 | 14.78±1.86 | 7.43±1.43 | 1.99±0.51 |
| HFD-CRP KO | 60.32±11.15 | 17.03±2.01 | 0.92±0.24 | 14.62±1.78 | 7.01±1.03 | 2.08±0.48 |
| HFD-DHEA-WT | 81.06±11.01^*^ | 12.02±1.73^*^ | 2.12±0.28^**^ | 20.00±2.30^*^ | 5.49±1.06 | 3.64±1.15^**^ |
| HFD-DHEA-CRP KO | 76.82±13.65^#^ | 11.03±2.28^#^ | 1.99±0.31^##^ | 19.21±2.55^#^ | 5.61±0.90 | 3.42±0.93^##^ |

ND, normal chow diet; HFD, high fat diet; DHEA ,dehydroepiandrosterone. E2, estradiol; P, progesterone; T, testosterone; LH, luteinizing hormone; FSH, follicle stimulating hormone. Data are expressed as means ± SD, ^*^*P*<0.05，^**^*P*<0.01 vs ND-WT group，^#^*P*<0.05，^##^*P*<0.01 vs ND-CRP KO group

**Supplementary Table 2. Blood pressure and [heart rate](javascript:;) in [different](javascript:;) groups**

|  | SBP (mmHg) | DBP (mmHg) | HR (bpm) |
| --- | --- | --- | --- |
| ND-WT | 104.20 ±5.12 | 70.06 ±5.80 | 429.72 ±28.01 |
| ND-CRP KO | 100.12 ±5.98 | 66.39 ±5.52 | 430.17 ±21.54 |
| ND-DHEA-WT | 119.30 ±8.99^*^ | 83.04 ±6.69^*^ | 425.00 ±24.46 |
| ND-DHEA-CRP KO | 106.06 ±8.07 | 70.03 ±6.33 | 426.17 ±16.30 |
| HFD-WT | 121.00 ±6.66 | 102.03 ±7.10 | 430.94 ±26.05 |
| HFD-CRP KO | 107.21 ±8.44^#^ | 78.23 ±6.82^#^ | 428.06 ±23.33 |
| HFD-DHEA-WT | 131.03 ±6.80 | 110.02 ±7.60 | 432.33 ±24.19 |
| HFD-DHEA-CRP KO | 112.03 ±7.31^a^ | 82.23 ±6.25^a^ | 423.00 ±19.73 |

ND, normal chow diet; HFD, high fat diet; DHEA,dehydroepiandrosterone. SBP, systolic pressure; DBP, diastolic blood pressure. HR, heart rate. Data are expressed as means ± SD, ^*^*P*<0.05 vs. ND-WT group, ^#^*P*<0.05 vs. HFD-WT group, ^a^*P*<0.05 vs HFD-DHEA-WT group

**Supplementary Table 3. Plasma metabolic parameters under basal and clamped conditions during central leptin infusion.**

|  | Insulin (mIU/ml) | Glucose (mmol/l) | TG  (mmol/l) | TC  (mmol/l) | FFA (mmol/L) |
| --- | --- | --- | --- | --- | --- |
| ND-DHEA-WT | | |  |  |  |
| Basal | 35.71 ± 11.38 | 6.0 ± 0.3 | 0.71 ± 0.14 | 1.68 ± 0.38 | 1.24 ± 0.27 |
| Clamp | 110.68 ± 16.36^＆^ | 5.8 ± 0.4 | 0.55 ± 0.11 | 1.16 ± 0.28^＆^ | 0.67 ± 0.14^＆^ |
| ND-DHEA-CRP KO | | |  |  |  |
| Basal | 29.80 ± 6.00 | 5.5 ± 0.5 | 0.67 ± 0.15 | 1.58 ± 0.36 | 1.21 ± 0.18 |
| Clamp | 95.12 ± 11.88^＆^ | 5.3 ± 0.2 | 0.38 ± 0.09^＆^ | 1.05 ± 0.28^＆^ | 0.57 ± 0.14^＆^ |
| HFD-DHEA-WT | | |  |  |  |
| Basal | 46.34 ± 8.02^*^ | 6.5 ± 0.7 | 1.12 ± 0.35^*^ | 2.38 ± 0.39^*^ | 2.00 ± 0.34^*^ |
| Clamp | 141.21 ± 16.06^＆^ | 6.3 ± 0.5 | 0.69 ± 0.09^＆^ | 1.40 ± 0.26^＆^ | 1.32± 0.13^＆^ |
| HFD-DHEA-CRP KO | | |  |  |  |
| Basal | 38.32 ± 8.39^#^ | 6.1 ± 0.5 | 1.02 ± 0.35 | 2.17 ± 0.35 | 1.98± 0.30 |
| Clamp | 125.15 ± 22.99^＆^ | 5.9 ± 0.2 | 0.53 ± 0.08^＆^ | 1.36 ± 0.39^＆^ | 1.09± 0.17^＆^ |

ND, normal chow diet; HFD, high fat diet; DHEA, dehydroepiandrosterone. Data are expressed as means ± SD, ^*^*P*<0.05 vs. ND-WT group, ^#^*P*<0.05 vs. HFD-WT group, ^＆^*P*<0.05 vs Basal

**
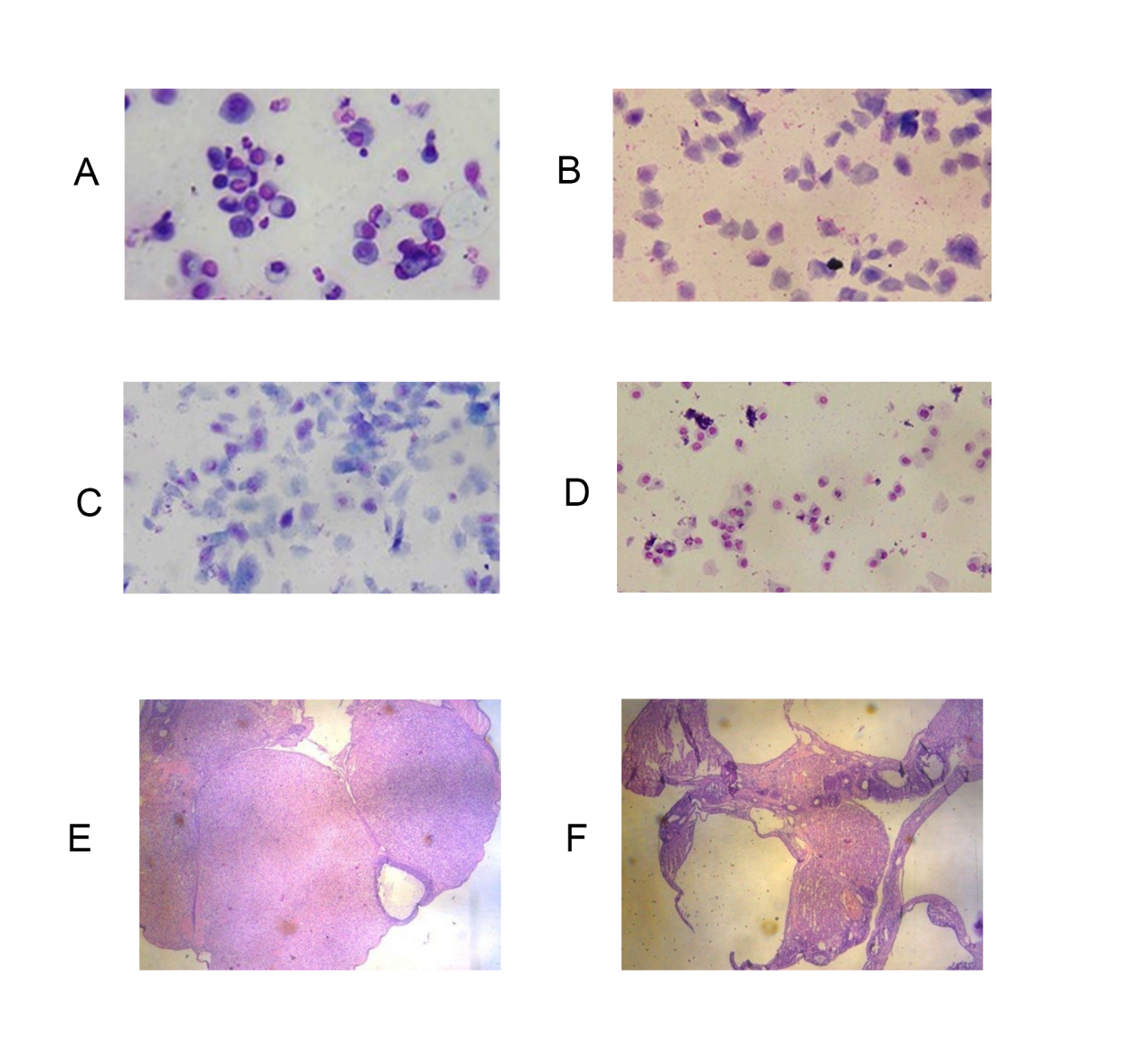
Supplementary** **Figure 1.** Smears of rat vaginal secretions (×20) and pathological morphology of ovarian tissues(×40) analyzed by light microscope. Estrous cycle monitoring (A) Proestrus. Oval nucleated epithelial cells are the majority, white blood cells and keratinized epithelial cells are rare. (B) Estrus. Lamellar keratinized epithelial cells predominate. (C) Late oestrus. Keratinized epithelial cells, nucleated epithelial cells, and white blood cells were all seen. (D) Anoestrus. White blood cells account for the vast majority (E) The ovaries of normal control rats showed multiple luteal bodies and follicles of different developmental stages, and the granule cells were multilayered in the follicles. (F) The number of follicles with cystic dilatation and the number of granulosa cell layers decreased in the ovaries of rats in the PCOS model group.
